# Network-based Observability and Controllability Analysis of Dynamical Systems: the NOCAD toolbox

**DOI:** 10.12688/f1000research.19029.2

**Published:** 2019-09-18

**Authors:** Dániel Leitold, Ágnes Vathy-Fogarassy, János Abonyi

**Affiliations:** 1 Department of Computer Science and Systems Technology, University of Pannonia, Egyetem u. 10, Veszprém, 8200, Hungary; 2 MTA-PE Lendulet Complex Systems Monitoring Research Group, University of Pannonia, Egyetem u. 10, POB. 158, Veszprém, 8200, Hungary

**Keywords:** Dynamical systems, Complex networks, Controllability and observability analysis, Robustness, MATLAB toolbox

## Abstract

The network science-based determination of driver nodes and sensor placement has become increasingly popular in the field of dynamical systems over the last decade. In this paper, the applicability of the methodology in the field of life sciences is introduced through the analysis of the neural network of Caenorhabditis elegans. Simultaneously, an Octave and MATLAB-compatible NOCAD toolbox is proposed that provides a set of methods to automatically generate the relevant structural controllability and observability associated measures for linear or linearised systems and compare the different sensor placement methods.

## Introduction

In the life sciences, the determination of driver nodes in networks that play a significant role in the emergence or treatment of diseases is an intensively researched field ^1^. The importance of determining the proper driver nodes, i.e. the ones that ensure physically feasible controllability with the minimum cardinality and energy requirement, in biological networks, or more generally in any dynamical system, is unequivocal, and the amount of research concerning network science has increased rapidly. A detailed study of the control principles in biological networks has already been published ^2^. A review about the utilisation of the network science-based determination of driver nodes has also been published that introduced the results of the analysis of the protein-protein interaction (PPI) networks, Caenorhabditis elegans neuronal network, neurochemical rat brain network, Saccharomyces cerevisiae cell cycle networks, Epithelial Mesenchymal Transition (EMT) network, myeloid differentiation regulatory network and Th differentiation network, moreover, the identification of drug targets was also presented ^3^.

The network science-based analysis of dynamical systems has spread rapidly as it provides simple and efficient tools to analyse the structural controllability of any linear or linearised system ^1^. In terms of controlling the human signalling network, the role of different proteins was also systematically analysed with the toolset of network controllability in 4 to highlight the role of cancer-associated genes. Target control with objective-guided optimisation (TCO) was introduced to control a set of variables (or targets) of interest while the number of drivers and constrained nodes were minimised and maximised, respectively. This method is capable of determining the leading phenotype transitions in biological networks that can be identified as drug targets ^5^. In large-scale human liver metabolic networks (HLMN), the driver metabolites have essential functions, moreover, the role of transport reactions and extracellular metabolites in terms of controlling HLMN have revealed the importance of the environment of human liver metabolism with regard to the health of the liver ^6^. Using statistical analysis, a subset of critical control nonprotein-coding RNAs (ncRNAs) enriched by human disease can also be determined ^7^. In intra-cellular networks, to understand the information flow, a natural control system was utilised and the robustness of such a control was analysed ^8^.

The contribution of this paper is to introduce the novel toolbox, NOCAD ^9^, and its applicability in the life sciences through the example of the local network of 131 frontal neurons of Caenorhabditis elegans ^10^. The proposed toolbox is also suitable for the comprehensive analysis of any linear or linearised dynamical systems through their static network representation ^11– 13^. Although in the literature the phrase dynamical network is commonly used, it does not mean that the nodes or connections are temporal but refers to the network of dynamical systems. In the nonlinear case, the methodology needs further clarification because for small nonlinear examples the results can be incorrect ^14^ and the cardinality of the assigned sensors underestimated ^15^. As a result, this toolbox deals with only the linear case, nonlinear system-related methods will be implemented later. In the following sections, the representation of linear systems as well as their structural controllability and observability are introduced. Then the theoretical background of the methodology is presented and the implemented functions and measurements introduced through the network of rostral ganglia of C.elegans.

## Existing software

Although considerable research has utilised this method ^16^, a flexible software tool which may be used to support research in this field has yet to be designed. Parallel studies have resulted in a collection of applications, toolboxes, plug-ins and scripts that analyse and determine several structural properties of genes, protein-protein interactions and even social or urban networks. Most of these applications only analyse the structural properties of static networks and just a handful of them utilise these structural properties to draw conclusions concerning the dynamics of the system investigated. As our toolbox belongs to the second group, in the following section, the available applications and programs of this group are elaborated on.

A brief summary of the available tools with expanded functionalities is given in Table 1. Applications or software packages implemented in Python and capable of analysing the controllability and observability of dynamical systems are: graph-control ^17^ and WDNfinder ^18^. The advantage of Python-based development lies in its widespread use and the countless methods and packages implemented in this language, including the tools developed for network analysis ^19^. Although in Python the focus is on developing a broad software package for complex systems analysis, this has yet to be fulfilled and all of the available solutions have limitations. The graph-control toolbox only analyses the impact of network topology on the number of inputs and implements the fast matching algorithm ^20^. Even though WDNfinder only determines the minimum driver node set (MDS) and classifies nodes based on MDS, it is incapable of facilitating extended analysis.

**Table 1.** Toolboxes that implement some functions for the dynamical analysis of complex systems based on their structural analysis.

Software	Language	Applied on	GUI	Ref.	Last updated
netctrl	C++	General networks	No	21	January 8, 2015
CONTEST	MATLAB	General networks	No	22	February, 2009
CytoCtrlAnalyser	Java	Biomolecular networks	Yes	23	May 25, 2017
graph-control	Python	General networks	No	17	December 16, 2015
WDNfinder	Python	Biological networks	No	18	June 24, 2018
enaR	R	Ecological networks	No	24	May 18, 2018

Additionally, the CytoCtrlAnalyser ^23^ plug-in for Cytoscape ^25^ has been developed, which was implemented in Java and offers graphical user interfaces as well. It evaluates control centrality, control capacity and classifies nodes for biomolecular networks. Furthermore, the Ecological Network Analysis in R software package (enaR) provides some dynamical analysis functions and can generate models to analyse ecological networks in the R environment ^24^. As can be seen, both software packages deal with special kinds of networks. The netctrl program can determine the driver nodes and switchboard dynamics model for any complex network ^21^. CONTEST is a MATLAB toolbox which can analyse the dynamics of complex systems, but these dynamics do not cover the structural controllability and observability properties ^22^ of the analysed system. Although the presented software packages ensure the design of a controllable and observable system, they do not provide the opportunity to analyse the designed system exhaustively. These functions are helpful in terms of supporting the work of experts, but are insufficient for the sophisticated analysis of systems.

## Methods

In the background of the toolbox the linear systems and their structural controllability and observability properties are stood ^26^. A linear time-invariant (LTI) system is commonly described by its state-space representation that consists of the state equation ( Eq. 1) and the output equation ( Eq.2).

$$\dot{x}=\text{A}x+\text{B}u\;(1)$$

y=Cx+Du(2)

In the state-space representation, ***x*** stands for state variables, ***u*** represents the inputs, i.e. the actuators, and ***y*** denotes the vector of outputs, i.e. the sensors of the system. Matrices **A** and **B** define how state variables and inputs influence changes to the state variables, while matrices **C** and **D** define how state variables and inputs influence the outputs, respectively. The cardinality of state variables, inputs and outputs are noted by *N*, *M* and *K*, respectively.

A dynamical system is said to be controllable if it can be driven from any initial state to any desired final state within a finite time with properly selected inputs. Observability is the mathematical dual of controllability. A system is said to be observable if its state can be determined at a given time by a finite set of measured input and output variables. Kalman’s rank criterion ^27^ was used to determine the structural controllability and observability as follows: if the rank of the controllability matrix is equal to the number of state variables, *rank*() = *N*, then the system is structurally controllable, where = [ **B, AB *, … ,* A ^N–1^*B***]. Analogously, if the rank of the observability matrix is equal to the number of state variables, *rank*() = *N*, then the system is structurally observable, where = [ **C** ^T^, ( **CA**) ^T^, … , ( **CA ^N–1^**)T] ^*T*^.

To ensure controllability (or observability) using a minimum number of inputs (or outputs), a brute force approach should generate 2 ^*N*^ – 1 configurations of matrix **B** (or **C**). To solve this challenging task, the maximum set of disjoint edges is generated by the maximum matching algorithm ^1^. Two edges are disjointed if they do not share a common starting point or endpoint. The matched nodes are the endpoints of the edges that are a member of the maximum set of disjoint edges, the others are unmatched. Then the unmatched nodes that are generated based on **A** are the sensor nodes, where outputs should be placed to grant structural observability, while the unmatched nodes generated based on **A** ^T^ are the driver nodes, where inputs should be placed to grant structural controllability. **A** ^T^ is also the adjacency matrix of the network representation that is the input of the toolbox. It is very important to note that the result of maximum matching is not unique, and it is possible that the matching is perfect, i.e. no unmatched nodes have resulted. In our implementation, the canonical decomposition of Dulmage-Mendelsohn was utilised to calculate maximum matching ^28^.

**Figure 1.**
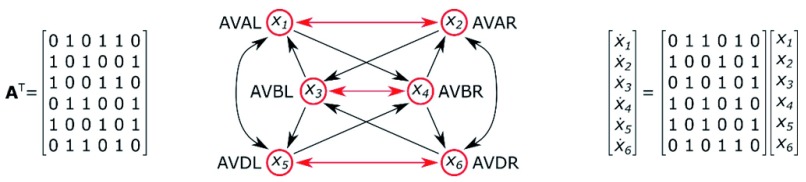
Representation of the command interneurons as a linear dynamical system. The adjacency matrix of the command interneurons, their network representation and the state equation without assigned input. In this example, due to the symmetric edge pairs between the nodes, the matching is perfect, i.e. all the nodes are matched. In this case, structural controllability and observability can be granted by selecting any node as a driver node and any node as a sensor node.

For a better understanding, we illustrate the aforementioned definitions by a small example in Figure 1 that contains the command interneurons AVAL, AVAR, AVBL, AVBR, AVDL and AVDR from the frontal neural network of neurons and synapses in C. elegans.

**Figure 2.**
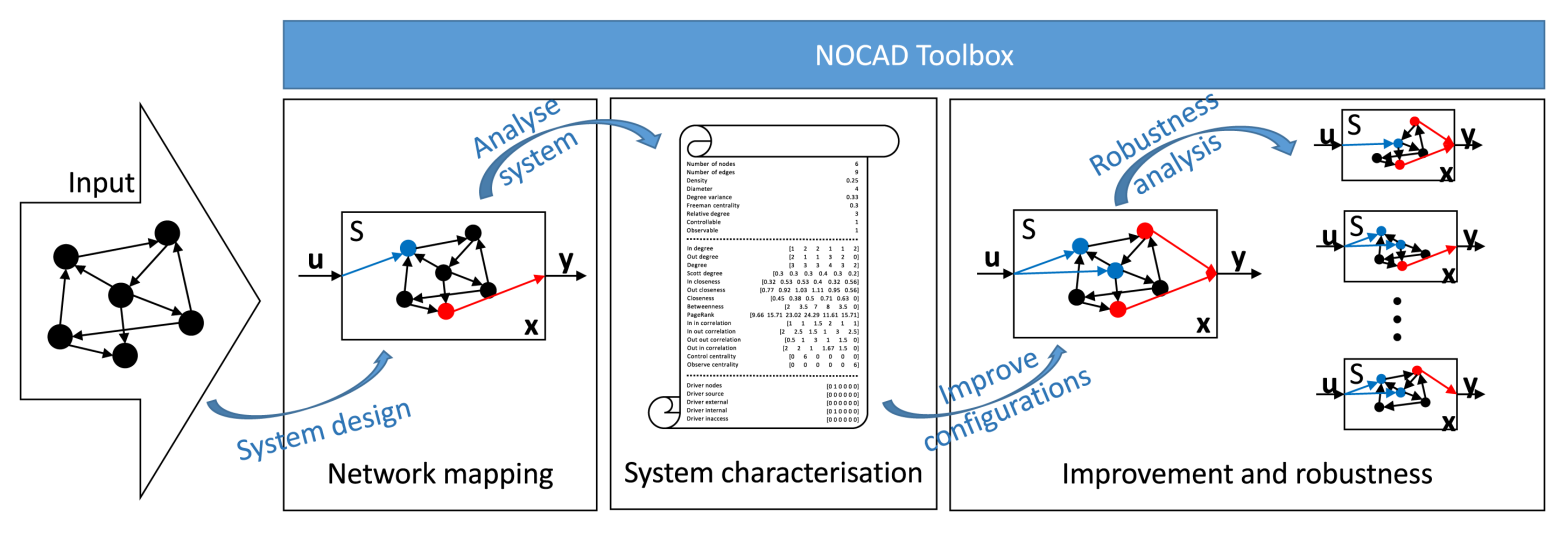
Workflow of the utilisation of the NOCAD toolbox. The network mapping module provides two methods to create a dynamical system based on the topology of the state variables. The system characterisation module generates more than 49 measures to analyse, classify and characterise the developed system. The improvement and robustness module offers five algorithms to improve the system with additional inputs (observers) as well as outputs (controllers) and can analyse the robustness of the designed system.

With the help of the presented Octave- and MATLAB-compatible toolbox, experts can create, analyse and improve any type of dynamical systems. As the structure of the dynamical systems is generally represented by their adjacency matrix and linear dynamical systems can be described by the state-space model that contains the dynamical, input, output and feedthrough matrices, the Octave/MATLAB programming language is a perfect environment to handle these matrices and provide comprehensive functionalities based on them. With the use of NOCAD ^9^, experts and researchers can effectively determine the input and output matrices of state-space models, calculate system-specific qualitative measurements (e.g. diameter, relative degree, control centrality and robustness of the system, etc.) and improve the system to satisfy the relative degree-based requirements. The workflow of the toolbox can be seen in Figure 2.

### Implementation

According to the aforementioned approach, the implemented functions of the toolbox were divided into three modules as follows: (1) network mapping module, (2) system characterisation module and (3) improvements and robustness module. The input of the first module is the adjacency matrix of the network to be analysed. The second module requires the matrices of the dynamical system generated by the first module. The result of the second module is a structure that is also the input of the third module.

The *network mapping* module creates a dynamical system from a given network structure, i.e. the necessary matrices of the state-space model are generated for the topology in such a way, that the created system is structurally controllable and structurally observable. The determination of the input and output matrices can be achieved by the path finding and signal sharing methods ^11^, which modify the result of the maximum matching algorithm.

The *system characterisation* module performs the calculation of 49 numerical measures to qualify the dynamical system based on its structure. The implemented measures, on the one hand, are well-known static measures (e.g. the number of nodes and edges, closeness and betweenness centralities), and, on the other hand, measures that characterise the dynamics of the system (e.g. structural controllability, observability, control centrality and relative degree). This module can also be used for the purpose of simple network analysis.

The *improvement and robustness module* integrates two main functions. On the one hand, it enables the input and output configurations of the system to be extended in such a way that the relative degree of the modified system does not exceed the initially defined threshold. For this purpose, this module implements five methods, namely the set covering-based grassroot and retrofit methods ^12^, the centrality measures-based method ^12^, the modified Clustering Large Applications based on Simulated Annealing algorithm (mCLASA), and the Geodesic Distance-based Fuzzy c-Medoid Clustering with Simulated Annealing algorithm (GDFCMSA) ^12, 13^. On the other hand, this module allows users to examine the robustness of the extended configurations by removing nodes from the network representation and by checking the structural controllability and structural observability of the damaged system.

Although the last module seems to be out of line at first, its existence is reasonable. The importance of the controllability of a complex system has already been addressed ^1^. In terms of control theory, the relative degree is an important measure to describe how fast the system can be influenced or how sluggish it is. In the field of biology, this “speed” is also important, e.g. the time elapsed between taking a painkiller and feeling its effect. The implemented methods are introduced in detail in the cited articles and the manual of the NOCAD toolbox.

### Operation

In order to use the NOCAD toolbox ^9^, installation of Octave or MATLAB is required. Then the directories of the toolbox must be copied into the working directory, or the directories of the toolbox must be added to the paths. The functions were implemented in Octave 5.1.0 and MATLAB R2016a on a Windows 64-bit system. On other operating systems, or with other Octave or MATLAB versions, proper operation is not guaranteed. Our toolbox is independent of other MathWorks toolboxes, it uses only the octave-networks-toolbox ^29^ and the greedy set covering implementation ^30^.

## Use cases

In this section, the main functionalities of the NOCAD toolbox ^9^ are presented through the analysis of the local network of 131 frontal neurons of Caenorhabditis elegans. The first step in the workflow is to create a state-space model based on the adjacency matrix that presents the structural description of the system that, in this case, has the size of 131×131 according to the 131 frontal neurons.

Two methods, path finding and signal sharing are proposed that were implemented to correct the insufficient result of maximum matching. Both methods are modified versions of the maximum matching algorithm. The maximum matching method determined the following 12 neurons to be driver nodes: RMEL, RMER, SIADL, SIADR, SIAVL, SIAVR, SIBDL, SIBDR, SIBVL, SIBVR, SMDDR and URYDR, moreover, determined 12 sensor nodes that correspond to the following neurons: AINL, ASHL, ASIR, ASJR, AWAL, IL2DL, IL2DR, IL2L, SIBDL, URBL, URBR and URYDL. As no critical strongly connected components were present, the results were identical in the case of both the path finding and signal sharing methods.

After utilising the second module of the toolbox, the measures that qualify the whole network with one value are introduced, as presented in Table 2. The network contains 131 neurons and 764 synapses. The density shows that the number of edges is less than a twentieth of the possible maximum, and the diameter of the system, namely the longest shortest path in the network that presents its structure, is 9. The degree variance is 44.3299 which is relatively high given the size of the network, while the Freeman’s centrality is 0.2057. The relative degree of the system is also 4. The Pearson correlation coefficient shows that the in-in, in-out and out-out correlations are slightly assortative in nature, while the out-in correlation is likely to be disassortative. The system is controllable and observable. As no loop is present in the network, the percentage of loops relative to edges is 0%. As 77 symmetrical connections are present between 687 connected node pairs, the percentage of the symmetric edge pairs is 11.2082%.

**Table 2.** Centrality measures of the system generated for the neural network of C. elegans.

Measure	Value
controllability	1
observability	1
number of nodes	131
number of edges	764
density	0.0445
diameter	9
Freeman's centrality	0.2057
degree variance	44.3299
relative degree	4
Pearson in-in	0.0426
Pearson in-out	0.0048
Pearson out-out	0.1694
Pearson out-in	-0.1524
percentLoops	0
percentSym	11.2082

The second module generates node centrality measures that can reveal structurally important nodes. Since the generated measures can be presented by large tables, they are attached in Excel format to the toolbox ^9^. This analysis shows that one of the most important values is the highest degree of the nodes, which belongs to RIAR, an interneuron located in the nerve ring ^31^. As Scott’s centrality is a normalised degree, the most important node is once again RIAR. The closeness of node *x _i_* is calculated as the ratio of the number of nodes reachable from *x _i_* to the sum of their distances from *x _i_* . The higher value indicates the more central position of the node, and now RIAL is the most central element. The betweenness centrality shows how many shortest paths intercept the given node. If a node has a high value, then it is a critical node in the structure. The highest value belongs to neurotransmitter RIH that is a serotonin ^32^. The PageRank assigns a percentage value to each node, based on their centrality roles if Markov-chains are modelled. The measure referred to as correlation shows the proportion of the number of edges of neighbours’ and the number of neighbours. This information is useful when determining the assortativity of the system. The control centrality and observe centrality measures determine how many state variables can be influenced or observed by the nodes.

The determined driver and sensor nodes can be classified into four groups ^33^. According to these groups, four phenomena can provide driver or sensor nodes. Firstly, *source nodes* when the node has no incoming edges, thus, a dedicated input is needed. Secondly, dilation, when the generated set of child nodes has higher cardinality than the number of parent nodes. A distinction is made between *internal dilation* and *external dilation*, in the former the child node is not a leaf, i.e. it has children, while in the latter the child is a leaf node, i.e. it has no children. The last type is the *inaccessible nodes* when the node has an incoming edge and no dilation is present, but the node is not reachable by a directed path from any of the inputs. These types are important properties, e.g. the existence of dilation or inaccessibility is detrimental to complete structural controllability ^3^. The controlling and observing matrices are sparse matrices as only the columns of drivers and sensors contain nonzero values. The values show the number of derivations necessary to influence or observe a state variable in the system. Next, the similarity of the driver and sensor nodes is presented. This similarity is based on how similar the set of nodes is, which can be reached for driving or observing. Furthermore, the necessary derivation to influence or observe them is also part of the comparison. *R _c_* and *R _o_* are the simple reachability matrices. They show which nodes can be controlled or observed by a given node in its structural meaning, i.e. the existence of a directed path between the nodes is shown. In *R _c_*, the *i ^th^* column shows which nodes can control node *i*. From the other viewpoint, elements in row *i* highlight those nodes which can be controlled by node *i*. It is very important that *R _c_* is only a reachability matrix, the structural controllability of the reachable nodes is not granted by a node that can reach them, but in some cases the structural controllability problem can be reduced to a reachability problem ^34^. The *R _o_* matrix can be interpreted analogously with regard to observability.

Finally, measures of edge centrality are generated by the system characterisation module. The betweenness has the same meaning as in the case of nodes, that is, it yields the number of shortest paths that intercept the edge ^35^. From this perspective, the most critical synapsis is the one between the command interneuron AVAL and amphid ADLL with a value of 640.5833. The endpoint similarity shows how similar the influenced and observed sets of the state variables with regard to the endpoints of edges are. This metric has a high value if the edge is part of a cycle or creates a bridge in the network. As no bridges are present in this network, only cycles can be recognised by this measure. The edge similarity shows how similar the roles of edges are, and it allows redundancies, to be located.

For the demonstration of the last module, four plus one methods were applied to the neural network of C. elegans. The set covering-based grassroot method (SetCovGr) optimises the placement of driver nodes and sensor nodes to provide an initially demanded relative degree, but this method does not take into account the original input and output configurations also, thus, structural controllability and observability is not granted also. The other four methods grant controllability and observability by expanding the minimal configurations. They are the centrality measures-based (CentMeas) retrofit, set covering-based retrofit (SetCovRet), modified Clustering Large Applications based on Simulated Annealing (mCLASA) and Geodesic Distance-based Fuzzy c-Medoid Clustering with Simulated Annealing algorithm (GDFCMSA) methods ^13^. These methods were utilised with the following parameters: the required relative degree was set at 2, while the alpha parameter of the cost function was set at 0.5 ^13^. The results can be seen in Table 3. The number of assigned driver nodes varies significantly when different methods are applied. The centrality measures-based method assigned the most driver nodes to the system. Thus, this method results in the smallest cost, but the difference is irrelevant, most of the methods resulted in a cost of 1.5. The increase of the number of the driver nodes decreases the mean relative degree, which is the lowest in the case of the centrality measures-based method.

**Table 3.** Improved input and output configurations for the neural network of C. elegans with the required relative order of 2.

	CentMeas	SetCovRet	SetCovRet	mCLASA	GDFCMSA
number of drivers	27	17	16	21	19
cost	1.4580	1.5610	1.5496	1.5382	1.5649
relative degree	2	2	2	2	2
mean of rel. deg.	0.9160	1.1221	1.0992	1.0763	1.1298
input robustness	117	116	116	120	117
input robustness (%)	0.8931	0.8855	0.8855	0.9160	0.8931
number of sensors	23	16	12	19	20
cost	1.4924	1.5687	1.6336	1.5382	1.5267
relative degree	2	2	2	2	2
mean of rel. deg.	0.9847	1.1374	1.2672	1.0763	1.0534
output robustness	121	121	120	121	121
output robustness (%)	0.9237	0.9237	0.9160	0.9236	0.9236

The robustness of the configuration was also analysed. In each scenario, a node was removed from the network. Using the leave-one-out strategy, the network with the altered configuration remains controllable in 115 scenarios. As for the sensor nodes, the difference is not as significant between the methods as in the case of the driver nodes. Critical nodes were also generated. A node is critical if the system becomes uncontrollable or unobservable if the node is removed. The determined critical nodes and the names of selected driver and sensor nodes can be found in the Excel file attached to the toolbox.

## Conclusions

Although numerous papers have utilised the network-based determination of driver and sensor nodes, a flexible toolbox that may be used to support the analysis has yet to be designed. To fill this gap, in this article the Octave- and MATLAB-compatible NOCAD toolbox ^9^ was proposed to support the network-based controllability and observability analysis of dynamical systems, and through the analysis of the neural network of C.elegans, the applicability of the toolbox in the life sciences was presented. The toolbox offers two methods to design a structurally controllable and observable system based on the adjacency matrix ( **A** ^T^). The designed system can be analysed by 49 qualitative measures both from structural and dynamical points of view. The toolbox serves five methods to improve the designed system by adding new inputs and outputs to it, thus, its relative degree can be decreased. Then the robustness of the individual designs can also be evaluated. The modular structure of the toolbox supports the facile improvement of the modules by adding new functions and the toolbox can be extended by new modules as well. Even though the modules are built on each other, most of their functions can also be used independently from each other.

Although our goal in this paper is to draw the attention of researchers of life sciences to the services provided by the NOCAD toolbox, it can be utilised in practice in various fields of sciences as well, for example, it enables social networks to be controlled in the economy, transaction networks to be analysed in finance or dynamical systems to be designed in engineering.

## Data availability

All data underlying the results are available as part of the article and no additional source data are required.

## Software availability

**Source code available from:** https://github.com/abonyilab/NOCAD.

**Archived source code at time of publication:** https://doi.org/10.5281/zenodo.2656674 ^9^

**License:** GNU General Public License v3.0
