# Supplementary material for: Network-based Observability and Controllability Analysis of Dynamical Systems: the NOCAD toolbox
Source: F1000Res. 2019 Sep 18;8:646. Originally published 2019 May 9. [Version 2] doi: 10.12688/f1000research.19029.2 (PMC6777013; doi:10.12688/f1000research.19029.2)
Supplement: Peer review history

# Peer review: Reviewer response for version 2

**DOI:** 10.5256/f1000research.22645.r54089

**Published:** 2019-10-03

**Authors:** Luis A. Aguirre, Arthur Montanari, Ercílio Moreira

**Affiliations:** 1 Graduate Program in Electrical Engineering, Federal University of Minas Gerais (UFMG), Belo Horizonte, Brazil; 2 Graduate Program in Electrical Engineering, Federal University of Minas Gerais (UFMG), Belo Horizonte, Brazil

The revised version is clearly more focused and directed to researchers is life science, although its application is much wider. It has been shortened and some figure removed. Discussions are more objective and some technicalities corrected.

 Some more general discussions that have been suggested in the first review were simply added as assertions. This helps keep the paper compact, which is understandable.

 As a second notice, though, we still think that the third module could be discussed in more depth. The toolbox provides five (“four plus one”) methods to analyze the network robustness (from a structural controllability/observability point of view) and to place driver/sensor in such a way that the relative degree of the network system does not exceed a specified threshold. Although the relevance of the third module is now more well-justified, it is not yet clear what is the difference between these five methods. What are the pros and cons of each method? How can this be perceived in the studied neural network of *C. elegans*?

 We found no compatibility issues with Octave in this version.

 Minor problems: 

1. In the definition of the observability matrix there is a “^” missing. The last T within the square brackets should be a transpose.
2. “Since the generated measures can be presented by large tables, they are attached in Excel format to the toolbox [9].” Ref. [9] is a link to the old toolbox (in Zenodo website). It would be nice to link the site with current version (GitHub).

We confirm that we have read this submission and believe that we have an appropriate level of expertise to confirm that it is of an acceptable scientific standard.

# Peer review: Reviewer response for version 2

**DOI:** 10.5256/f1000research.22645.r54088

**Published:** 2019-09-30

**Authors:** Gilles Didier

**Affiliations:** 1 Institut Montpelliérain Alexander Grothendieck (IMAG), CNRS, University of Montpellier, Montpellier, France

Thanks for your revised manuscript which addressed most of my comments. In particular, the structure of the paper was improved and the notions used in your toolbox are now introduced. I also appreciated the new example which provides a better illustration of the possibilities of your package. Though I am not a native speaker myself, I feel that some issues remained in your text.  Please find below suggestions to improve the writing.

**Abstract:**

 I suggest the following alternative:

 "The network science-based determination of driver nodes and sensor placement has become increasingly popular in the field of dynamical systems over the last decade. We developed an Octave and MATLAB-compatible NOCAD toolbox which implements various methods to compute relevant structural, controllability and observability measures associated to linear or linearised systems and to compare different sensor placement methods.

 We illustrated the use of our toolbox in life sciences by applying it to the analyse the neural network of *Caenorhabditis elegans*."

**Introduction:**

 An alternative first paragraph:

 "In the life sciences, the determination of nodes that play a significant role in networks (e.g., in the emergence or the treatment of diseases) is an intensively researched field^1. In particular, determining the proper driver nodes, i.e. the ones that ensure physically feasible controllability with the minimum cardinality and energy requirement, in biological networks, or more generally in dynamical systems, is an important question.  We refer to 2 for a detailed study of control principles in biological networks. A recent review paper presents presents the application of the network science-based determination of driver nodes to the analysis of various biological networks (protein-protein interaction -PPI- networks, Caenorhabditis elegans neuronal network, neurochemical rat brain network, Saccharomyces cerevisiae cell cycle networks, Epithelial Mesenchymal Transition -EMT- network, myeloid differentiation regulatory network and Th differentiation network) and to the identification of drug targets^3."

 I am not sure to well understand your sentence: "This method is capable of determining the leading phenotype transitions in biological networks that can be identified as drug targets 5"

 Do you mean something like: "This method is capable of determining genes controlling phenotype transitions in biological networks, thus may help to indentify drug target candidates^5" ?

**Please reformulate:**

 "In large-scale human liver metabolic networks (HLMN), the driver metabolites have essential functions, moreover, the role of transport reactions and extracellular metabolites in terms of controlling HLMN have revealed the importance of the environment of human liver metabolism with regard to the health of the liver^6. Using statistical analysis, a subset of critical control nonprotein-coding RNAs (ncRNAs) enriched by human disease can also be determined^7 . In intra-cellular networks, to understand the information flow, a natural control system was utilised and the robustness of such a control was analysed^8".

 The paragraph is a bit confusing and the relation with your work is not obvious. I guess that the examples presented was analysed with the same approaches as those implemented in your toolbox but this should be stated somewhere.

**Find below, an alternative suggestion for the last paragraph. I am not sure that it is the right place for the second sentence.**

 "The aim of this paper is to introduce the novel toolbox, NOCAD^9 , and to illustrate its applicability in the life sciences through the analyse of the local network of 131 frontal neurons of Caenorhabditis elegans^10. The NOCAD toolbox is also suitable for the comprehensive analysis of any linear or linearised dynamical systems through their static network representation^11–13. 

 We emphasise that in our context, the expression "dynamical network" does not implies that the nodes or connections change with time but refers to networks arising from dynamical systems.

 The current version of NOCAD deals with only the linear case. Nonlinear system-related methods will be implemented later. In the nonlinear case, the methodology needs further clarification because for small nonlinear examples the results can be incorrect 14 and the cardinality of the assigned sensors underestimated^15 .

 Theoretical background and formal definitions of the representation of linear systems and of their structural controllability and observability are provided below."

**Other comments:**

- page 4 col 1 first paragraph "provide the opportunity" -> "allow".
- page 4 col 1 second paragraph "stood" -> "fixed".
- page 4 col 2 first paragraph "if the rank of the controllability matrix is equal to the number of state variables, rank(C) = N" -> "if the rank of the controllability matrix C is equal to the number of state variables, i.e., rank(C) = N" (same suggestion for the sentence about the observability matrix).
- page 4 col 2 "For a better understanding, we illustrate the aforementioned definitions by a small example in Figure 1 that contains the command interneurons AVAL, AVAR, AVBL, AVBR, AVDL and AVDR from the frontal neural network of neurons and synapses in C. elegans." -> "Figure 1 illustrates the aforementioned definitions with a small example which contains the command interneurons AVAL, AVAR, AVBL, AVBR, AVDL and AVDR of the C. elegans' frontal neural network."
- page 5 col 1 end of paragraph 1: "can be seen" -> "is displayed".
- page 5 col 2 par 2: remove the comma after "in such a way".
- page 6 col 1 last par.: your last sentence is too long. ", moreover," -> ". It also".
- page 6 col 2 last par.: "can be presented by" -> "are stored in".
- page 6-7: "The higher value indicates the more central position of the node, and now RIAL is the most central element." -> "The closeness of a node measures its centrality. In our dataset, RIAL is the most central element under this criteria."
- page 7 col 1 par 1: "if Markov-chains are modelled" -> "under Markov models".
- page 7 col 1 par 1: "the proportion of the number of edges of neighbours’ and the number of neighbours" -> "the proportion of the number of edges of neighbours to the number of neighbours".
- page 7 col 1 par 1: "when determining" -> "to determine".
- page 7 col 1 par 2: "source nodes when the node has no incoming edges, thus," -> "source nodes are nodes with no incoming edges, for which".
- page 7 col 1 par 2: "dilation, when" -> "dilation occurs when".
- page 7 col 1 par 2: "between internal dilation and external dilation, in the former the child node is not a leaf, i.e. it has children, while in the latter the child is a leaf node, i.e. it has no children." -> "between internal dilation nodes and external dilation nodes according to whether their child node is an internal node, i.e., has children, or a leaf, i.e., has no children, respectively."
- page 7 col 2 last par.: "Although numerous papers used the network-based determination of driver and sensor nodes, a flexible toolbox that may be used to support the analysis has yet to be designed. To fill this gap, in this article the Octave- and MATLAB-compatible NOCAD toolbox 9 was proposed to support the network-based controllability and observability analysis of dynamical systems, and through the analysis of the neural network of C.elegans, the applicability of the toolbox in the life sciences was presented." -> "Although numerous papers have utilised the network-based determination of driver and sensor nodes, a flexible toolbox implementing this approach has yet to be designed. To fill this gap, we propose the Octave- and MATLAB-compatible NOCAD toolbox 9 which performs the network-based controllability and observability analysis of dynamical systems. The applicability of the toolbox in the life sciences was demonstrated through the analysis of the neural network of C.elegans."

I confirm that I have read this submission and believe that I have an appropriate level of expertise to confirm that it is of an acceptable scientific standard.

# Peer review: Reviewer response for version 1

**DOI:** 10.5256/f1000research.20856.r48912

**Published:** 2019-06-25

**Authors:** Luis A. Aguirre, Arthur Montanari, Ercílio Moreira

**Affiliations:** 1 Graduate Program in Electrical Engineering, Federal University of Minas Gerais (UFMG), Belo Horizonte, Brazil; 2 Graduate Program in Electrical Engineering, Federal University of Minas Gerais (UFMG), Belo Horizonte, Brazil

In this paper the authors describe the NOCAD toolbox developed for the analysis of some aspects of networks as, for instance, structural controllability and observability. The topic of the paper is not only very interesting but also timely as issues relating to controllability and observability of networks could be fundamental in a number of practical situations. Hence to have a nice set of tools to analyze dynamical networks is welcome.

 The toolbox described is useful in a specific range of problems. Here we focus on the controllability and observability properties of networks, as prompted by the title. The tools presented focus on structural controllability and observability of linear systems. The assumption of linearity is not mentioned in the abstract. As a matter of fact, the opening phrase of the last paragraph in the introduction should be copied to the abstract.

 The nonlinear case and other definitions of controllability and observability, such as, dynamical and symbolical are not mentioned in the paper nor are handled by the toolbox. This is an important remark as it is now known that some algorithms underestimate the cardinality of the set of sensor nodes when applied to nonlinear systems.

**The paper**

 In the second paragraph of the Introduction, the authors mention the importance of “determining the proper driver nodes”. We wonder if the average reader would know what that is. In clarifying this point the authors would like to address first what is “a” proper set of driving nodes (e.g. one that will guarantee full controllability). However, in order to specify “the” proper set, possibly some more detailed measure of controllability should be employed. For instance, suppose two sets of driving nodes S1 and S2, both with the same cardinality, such that the network is fully controllable either from S1 or from S2. Hence S1 is “a” proper set of driving nodes (assuming that by proper the authors are referring to full controllability) and S2 is another one. Now, it could well be that using S1 less energy is required to drive the network from one state x(ti) to another x(tf) when compared to S2. In that case, S1 and S2 are not totally equivalent. Structural controllability and observability are unable on their own to provide this distinction. 

 Following the same vein, we wonder if the average reader of F1000Research would know the distinction of static and dynamic networks. A word about this would be profitable, especially because the toolbox refers mainly to the second class.

 In some parts of the paper the authors refer to matrix A as the “state transition” matrix. In continuous-time this is incorrect. In discrete-time this is only correct for a transition time of one sampling period. Matrix A is called the “dynamical matrix”. State transition matrix is something else.

**Relevance to the journal**

 The MS introduction is written in such a way that it instigates interest from the target audience of this journal (mathematical biology). However, the techniques implemented in the toolboxes are usually found around the network science community, which includes several other fields that range from power systems to social networks. To show more coherence to this journal, the authors should provide some example of application on a “real-world network” under a biological context. This is also interesting to show how the results provided by the toolbox can be useful to draw conclusions under a practical context other than a “toy problem” as presented.

**Background**

 The MS content is not sufficient to understand the techniques implemented in the toolbox. We know that the paper does not aim at providing extensive background, but some could be helpful. A reader coming from a control theory background might expect the toolbox to provide results based on Kalman’s definition of controllability and observability. However, the toolbox is based on Liu and coworker’s maximum matching algorithm which is based, in turn, on Lin’s structural definition (1974) (which is not even mentioned in the MS). Which definition of controllability and observability the toolbox is based on should be crystal clear in the main text and abstract. Moreover, the notions of structural controllability and observability should be presented to the reader and how they interplay with the more well-known notion in Kalman’s sense (e.g. Lin’s definition is only a necessary condition for Kalman’s definition).

 Although we think that the definitions of controllability and observability should be mentioned on the main text, the authors provide some background on the implemented maximum matching algorithms in the toolbox. This, however, should be mentioned explicitly in the MS.

**Examples**

 The paper furnishes examples to illustrate some of the features of the new toolbox. The network topology is the same in each example, which facilitates understanding and comparison. In some cases, more discussion would be welcome. For instance, in the last example (Figure 7) not all the indices are clearly defined to the user, for instance the critical nodes are given as x2, x4 and x7. From the context it seems that if any of these nodes is lost, the network would become uncontrollable, but this is not directly stated.

 In referring to Figure 5 the authors speak in terms of Reachability matrices: Rc and Ro. It is clear from Figure 5 that Rc shows which nodes have a path to node i (the ith column). As for Ro, it seems that we should look row-wise instead of column-wise, is that right? Anyhow, we do not think it is adequate to say that Rc shows which nodes can be controlled from another one. The word “control” is perhaps too general. We would suggest just to say that Rc shows which nodes can be reached from another one.

**Toolbox**

 The toolbox is subdivided in three modules. The first one, “network mapping module”, implements the maximum matching algorithm (and related modifications) to return a structurally controllable (observable) network with the smallest set of driver (sensor) nodes. This is a welcome feature, especially for a MATLAB environment, which we are unaware of any alternative.

 The second module, “system characterization module”, provides several graph measures that are available on other MATLAB-based toolboxes, but are indeed useful to assess the network controllability and observability properties of a system. Thus, its presence is justifiable.

 The third module, “improvements and robustness module”, is a set of functions which specifically implement previous results of the authors (e.g. Refs. 20 ^1^, 21 ^2^). It seems quite specific, but nevertheless the toolbox relevance is justifiable in great part for its module one.

 The toolbox seems fast and no bugs were found in its implementations as far as the MATLAB environment is concerned. See further comments on some compatibility issues when using Octave.

**The target audience of the toolbox**

 The toolbox is applicable to any kind of network (graph), be it directed or undirected, weighted or unweighted, and so on. However, although general, the authors should discuss in the MS what are the kinds of networks where a structural analysis of controllability and observability are more useful. For instance, a linearization of a power system model modelled by interconnected Kuramoto oscillators yield a dynamical matrix “A” whose corresponding adjacency graph is not only highly connected but also undirected. Consequently, the toolbox points out that only one driver node is needed to structurally control the network, independently of its size “n”(which is true in this case according to Lin’s definition). However, this does not give insight to the problem since basically any node can be chosen as a driver node, and only one node being sufficient seems quite unrealistic. The question is: For what kind of networks is the structural approach more interesting (and hence the toolbox)? It could be that the toolbox could be extra-helpful in the context of more sparse and directed networks (with higher hierarchy).

**Some nitpicking:**

 Sometimes it is not clear whether the authors refer to the dynamical matrix “A” or the adjacency matrix “A^T”. We recommend that different nomenclatures be used, such as “A” for the dynamical matrix and “A_{Adj}” for the adjacency matrix. This should be changed in the MS and manual.

 Regarding the installation of the NOCAD toolbox. We noticed that the NOCAD already comes with a “octave-network-toolbox” folder which does not have all the necessary functions to use Module 2. Thus, we had to download the “octave-network-toolbox-master” folder in Ref. 22 to have access to all needed functions. Is this necessary or is the NOCAD toolbox really missing some functions?

 Some statements in Section “Use Cases”, Paragraph 5, are redundant with the information already present in Fig. 4. Instead of repeating the same information, it might be more interesting to make some comments on how useful some of these network measures can be to design better and more robust networks from a control and observation point-of-view.

 There are some issues in the Octave version of this toolbox. For instance, function “heatmaps” has some bugs in Octave but works well in MATLAB. This happens because the function “colormap” from Octave accepts the argument “hot” but not “Hot”. The authors should do a careful review of the Octave toolbox and check for further compatibility issues.

We confirm that we have read this submission and believe that we have an appropriate level of expertise to confirm that it is of an acceptable scientific standard, however we have significant reservations, as outlined above.

## References

[1] : Evaluation of the Complexity, Controllability and Observability of Heat Exchanger Networks Based on Structural Analysis of Network Representations. *Energies*.2019;12(3) : 10.3390/en12030513 10.3390/en12030513

[2] : Network Distance-Based Simulated Annealing and Fuzzy Clustering for Sensor Placement Ensuring Observability and Minimal Relative Degree. *Sensors (Basel)*.2018;18(9) : 10.3390/s18093096 10.3390/s18093096 30223464PMC6164052

# Peer review: Reviewer response for version 1

**DOI:** 10.5256/f1000research.20856.r48269

**Published:** 2019-05-28

**Authors:** Gilles Didier

**Affiliations:** 1 Institut Montpelliérain Alexander Grothendieck (IMAG), CNRS, University of Montpellier, Montpellier, France

This software tool article presents the Octave/MATLAB toolbox "NOCAD" for networks analysis. NOCAD implements various standard measures on networks and their nodes (density, betweenness, centrality...). Its main novelty stands in the ``dynamical-systems-based'' tools to analyse (static) networks that it provides. This article can be seen as a companion paper of the reference 19 ^1^ from the same authors (It could be stated somewhere in the text).

 The ideas implemented in the toolbox are interesting, but I feel that their presentation has to be improved. First, I suggest you get your text proof-read by a native speaker. Second, many concepts used in the manuscript should be at least briefly recalled. Third, the article lacks a detailed description of the input (in the general sense, including user interaction) of the toolbox. The basic input seems to be the incidence matrix of a directed network but, if I well understood, the toolbox allows to set input and output nodes and has network design capabilities.

 Another major concern is that though one of the aims of the article is to draw attention of life science researchers, it does not present clearly how the toolbox can be used on biological networks and on which kind of biological networks it can be applied. Real biological examples should be actually more than welcome in the "Use cases" section, which contains only toy examples. Without such examples, I am afraid that the article fails to draw attention of its expected audience. In particular, I am wondering in what extent the toolbox can be used on standard biological datasets, e.g., PPI networks can have thousands vertices.

 Please find below some detailed comments:

**Abstract**

 The abstract has to be extended in order to give a better idea of the content of the article.

**Introduction**

 The introduction does not reflect well what follows and the topic of the paper. You should start by briefly introducing the general ideas of dynamical analysis of networks, precise in what sense you use the term "driver node", which is sometimes used in a biological context etc. First mention of dynamical system comes at the end of the second paragraph and could be developed (e.g. by briefly recalling ideas of [7] ^2^) while biological examples may not need to be as much detailed.

 The description of previous software sounds good. It may take place a dedicated section.

**Method**

 The method section should recall the notions of controllability and observability and the general principles of ``dynamical analysis'' (it can be brief, something like the two first subsections of the section "Results" of [19] ^1^). In the current version, I don't think that this part can be understood without reading your reference [19] ^1^.

 You should state explicitly which are the inputs of your toolbox, the accepted formats etc.

 I found the paragraph about the modularity of the toolbox is not very useful. It could be shortened or even discarded.

 Subsection "Operation" ("Installation"?) is OK.

**Use cases**

 If I well understood, the input is the incidence matrix A from which are computed the matrices B, C and D as illustrated with the first example. It should be interesting to apply the approach on a biological network, for instance a protein-protein interaction network or a regulatory network, in order to illustrate the interest of the results obtained in this context.

 In the second example (Figure 3), input and output nodes seem to be set arbitrarily in order to get a system complex-enough to show the function of the second module of the toolbox (incidence matrix is the same as that of the first example). Is it possible to illustrate the toolbox with an example in which the input and outputs are not fixed? I am actually concerned with the fact that the use cases do not really show how the toolbox can be used on a real dataset, which may not have natural input and outputs.

 Figures 4, 5 and 6 displays the results of the toolbox on the second example. These results are briefly discussed in the text which also recalls the definitions of some of the measures. Applying the toolbox on a real dataset would make the discussion of the results of the toolbox much easier to interpret. Moreover, a real dataset could provide evidence of the relevance of the results obtained by relying on the nature of nodes distinguished by the toolbox.

 The different types of nodes displayed in Figure 5 should be defined rather than just refer to [24] ^3^.

 Could you please give an idea about what kind of biological networks can be studied by the means of your third module? This part is not very intuitive from a biological point of view.

I confirm that I have read this submission and believe that I have an appropriate level of expertise to confirm that it is of an acceptable scientific standard, however I have significant reservations, as outlined above.

## References

[1] : Controllability and observability in complex networks – the effect of connection types. *Scientific Reports*.2017;7(1) : 10.1038/s41598-017-00160-5 10.1038/s41598-017-00160-5 28273948PMC5427891

[2] : Control principles for complex biological networks. *Brief Bioinform*.2018; 10.1093/bib/bby088 10.1093/bib/bby088 30239577

[3] : Control profiles of complex networks. *Science*.2014;343(6177) : 10.1126/science.1242063 1373-6 10.1126/science.1242063 24653036
